# Peroxisome Proliferator-Activated Receptor-γ as a Target and Regulator of Epigenetic Mechanisms in Nonalcoholic Fatty Liver Disease

**DOI:** 10.3390/cells12081205

**Published:** 2023-04-21

**Authors:** Mohamed Zaiou

**Affiliations:** Institut Jean-Lamour, Université de Lorraine, UMR 7198 CNRS, 54505 Vandoeuvre-les-Nancy, France; mohamed.zaiou@univ-lorraine.fr

**Keywords:** noncoding RNAs (ncRNAs), peroxisome proliferator-activated receptors, DNA methylation, histone modifications, hepatic steatosis

## Abstract

Peroxisome proliferator-activated receptor-γ (PPARγ) belongs to the superfamily of nuclear receptors that control the transcription of multiple genes. Although it is found in many cells and tissues, PPARγ is mostly expressed in the liver and adipose tissue. Preclinical and clinical studies show that PPARγ targets several genes implicated in various forms of chronic liver disease, including nonalcoholic fatty liver disease (NAFLD). Clinical trials are currently underway to investigate the beneficial effects of PPARγ agonists on NAFLD/nonalcoholic steatohepatitis. Understanding PPARγ regulators may therefore aid in unraveling the mechanisms governing the development and progression of NAFLD. Recent advances in high-throughput biology and genome sequencing have greatly facilitated the identification of epigenetic modifiers, including DNA methylation, histone modifiers, and non-coding RNAs as key factors that regulate PPARγ in NAFLD. In contrast, little is still known about the particular molecular mechanisms underlying the intricate relationships between these events. The paper that follows outlines our current understanding of the crosstalk between PPARγ and epigenetic regulators in NAFLD. Advances in this field are likely to aid in the development of early noninvasive diagnostics and future NAFLD treatment strategies based on PPARγ epigenetic circuit modification.

## 1. Introduction

Nonalcoholic fatty liver disease (NAFLD), which affects 25–30% of the global population, is currently one of the main public health and economic burdens [1]. Depending on the degree of liver abnormalities, the pathologic spectrum of NAFLD ranges from simple steatosis to its more aggressive form, nonalcoholic steatohepatitis (NASH), which can progress to advanced stages such as liver fibrosis, cirrhosis, and hepatocellular carcinoma (HCC) [2]. According to current knowledge, NAFLD is closely associated with several prevalent risk factors, including atherogenic dyslipidemia, type 2 diabetes mellitus (T2DM), metabolic syndrome, cardiovascular disease (CVD), and insulin resistance (IR) [3,4,5]. Epidemiology research shows that NAFLD is a metabolic disorder with a complex multifactorial pathogenesis and heterogeneous clinical manifestations [6], which varies between individuals with comparable lifestyles and metabolic abnormalities

Based on the heterogeneous aspect of this disease, an international panel of experts proposed a name change for NAFLD to Metabolic Associated Fatty Liver Disease (MAFLD) [7]. Although interesting, the newly proposed terminology has not been widely accepted and a debate on the subject is ongoing [8,9]. The pathogenesis of NAFLD is complicated and not well understood. Two hypotheses have been postulated to explain the onset and progression of this condition. The initial hypothesis representing the first “hit” is referred to as a “two-hit model”, in which liver fat accumulation and IR lead to liver damage in NASH [10,11]. Because NAFLD is well established as a multifactorial disorder in which multiple insults act together to induce pathology, the first hit may not be an accurate explanation of the pathogenesis of the disease. Therefore, scientists have turned to the second and increasingly accepted hypothesis, “multiple parallel hit”, which provides a more reliable explanation for the complex characteristics of NAFLD. This hypothesis suggests that, in addition to adipose tissue fat accumulation and lipotoxicity, other factors such as IR, mitochondrial dysfunction, increased endoplasmic reticulum stress, epigenetic alterations, and changes in the gut microbiota may act in concert in genetically predisposed individuals to promote liver inflammation and fibrosis [12,13,14].

There are limited therapeutic options and no approved drug that specifically targets NAFLD, despite intensive research on novel treatment for this condition and continuing clinical trials [15]. Lifestyle intervention, including diet and exercise, is still a preferred method. As IR is the most specific metabolic risk and pathologic hallmark of NAFLD, the use of PPARγ agonists, insulin-sensitizing thiazolidinedione (TZD) molecules, to treat NASH patients has also been investigated [16], as IR is the most specific metabolic risk and pathologic hallmark of NAFLD. However, the exploitation of peroxisome proliferator-activated receptor-γ (PPARγ) signaling in a therapeutic setting of NAFLD has been hampered by the limited understanding of its regulatory mechanisms and the lack of its precise function in liver-adipose tissue crosstalk.

**Figure 1:**
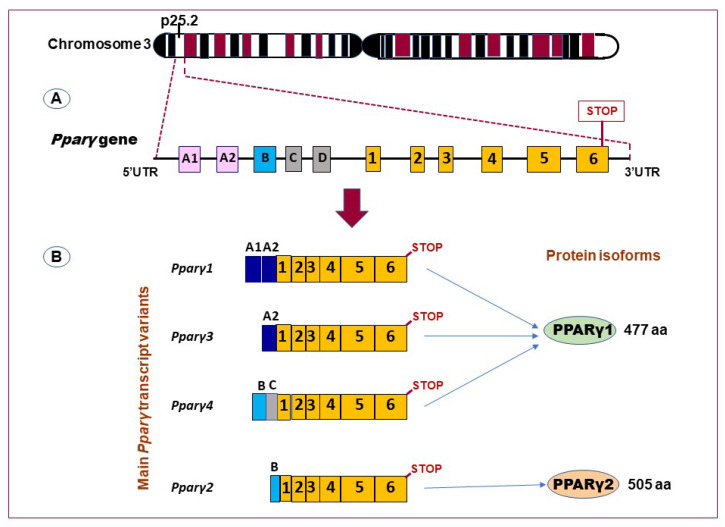
Schematic representation of the human *Pparγ* gene and its main transcript variants. (**A**) *Pparγ* gene lies on chromosome 23, band 3p25, and composed of at least 11 exons (exon A1–2, exon B–D, and exons 1–6). (**B**) Alternative promoter and mRNA splicing generate several variants (the transcript variants *Pparγ* 1,3, and 4 encode PPARγ1 isoform (477 amino acids; aa). The transcript variants *Pparγ2* encodes the PPARγ2 isoform (505 aa). (Adapted from reference [21]).

The superfamily of peroxisome proliferator-activated receptors (PPARs) has three subtypes: PPARα, PPARγ, and PPAR-β/δ, which regulate many metabolic pathways in a tissue-specific manner [17]. PPARγ was identified previously by Tontonoz et al. [18] and the corresponding gene, *Pparγ*, was mapped on chromosome 3p25.2 in humans [19]. The human *Pparγ* gene is composed of 9 exons exon A1, exon A2, exon B, and exons 1–6. This gene generates four *Pparγ* splice variants (*Pparγ* 1–4), which differ at their 5-end due to differential promoter usage and alternative splicing, and encodes for two protein isoforms (Figure 1) [20,21]. The PPARγ1 isoform, which is a 477 amino acid protein, is produced by the mRNAs *Pparγ1*, *Pparγ3*, and *Pparγ4*. The PPARγ2 isoform is translated from the *Pparγ2* mRNA transcript and has an extra 30 amino acids at its NH2-terminus [22]. 

PPARγ is involved in multiple physiological and pathological processes [23]. Based on the tissue in which it is expressed, PPARγ has three different isoforms. PPARγ2 expression is mostly restricted to adipose tissue, whereas PPARγ1 is found in nearly all cells [18,24,25]. It is also worth noting that, while PPARγ2 expression is induced in fatty liver, it remains substantially lower than in adipose tissue. On the other hand, macrophages, adipose tissue, and the colon are where PPARγ3 is most abundantly expressed [26]. The primary function of PPARγ is believed to be in adipose tissue, where it is known to induce adipocyte differentiation and promote triglyceride storage, hence reducing liver lipotoxicity and improving steatosis [27]. Transcription levels of this nuclear factor have been found elevated in the steatotic livers of obese individuals and animal obesity models [28,29]. Consistent with this finding, a different study found that PPARγ2 inactivation reduced the severity of fatty liver that had been induced by a high saturated fat diet in mice [30]. Furthermore, rosiglitazone and pioglitazone significantly reduced hepatic steatosis, as shown by clinical human studies [16,31,32]. There is growing evidence that the pathophysiology of NAFLD is significantly influenced by epigenetic changes, including altered DNA methylation patterns, posttranslational modifications of histones, and ncRNAs. However, it is yet unclear how these changes along with PPARγ influence the onset and progression of the disease.

## 2. The Role of Epigenetic Mechanisms in Regulating PPARγ Regulatory Function in NAFLD

Contrary to genetic changes in heritability, epigenetics is the study of heritable modifications in gene activity that do not involve direct alteration of the underlying DNA sequences [33]. Epigenetics determines the architecture of chromatin in cell nucleus, and therefore affects specific genomic sequences accessible to cellular regulatory machineries. The epigenome is susceptible to dysregulation throughout life but is highly vulnerable to environmental factors during fetal life since this is a period of rapid DNA synthesis [34]. 

Cells’ transcriptomes can be modified in response to both internal signals and environmental cues via epigenetic modulators, primarily DNA methylation, histone modifications, and alterations in ncRNAs expression patterns. As was already indicated, alterations in the liver epigenetic mechanisms have been demonstrated to have a role in the emergence of NAFLD [35] in part through crosstalk with PPARγ. Given the crucial role that PPARγ plays in lipid metabolism and lipogenesis, the identification of epigenetic changes underlying its regulation in the development of NAFLD is of special relevance for study in this field. In light of this, the next sections provide an overview of the existing literature on altered reciprocal regulation between epigenetic modulators and the PPARγ signaling pathways in NAFLD.

### 2.1. DNA Methylation

The most prevalent epigenetic mark in the mammalian genome is DNA methylation, which occurs when a methyl group is added to the C5 position of cytosine to form 5-methylcytosine. DNA methylation affects the accessibility of the transcriptional machinery to a DNA region that regulates gene expression. When confined to gene promoters, DNA methylation is often a repressive epigenetic signal [36]. The methylation of DNA bases, which is important for controlling the expression of imprinted genes, has been linked to a variety of human diseases, including NAFLD [37,38,39]. In patients with NAFLD, both hepatic DNA methylation and insulin resistance play a key role in the disease progression from simple steatosis to severe fibrotic NASH [40]. 

When comparing the DNA methylation levels of numerous genes in liver samples from NAFLD patients to those from healthy subjects, differences have been observed [41,42]. Even though PPARγ is less abundant in the liver than PPARα, it is still crucial for liver function, and the DNA methylation state of the *Pparγ* gene has been identified as a marker of the progression of liver disease. In a case-control study of NAFLD patients, increased hepatic methylation of the promoter of the PPARγ coactivator one-alpha (*PGC1-α*) gene, a key transcriptional regulator of mitochondrial fatty acid oxidation, significantly correlated with peripheral IR status and fasting insulin levels [43]. Furthermore, in human NASH liver biopsies, it has been shown that the promoter region of *Pparγ* undergoes methylation remodeling and becomes hypermethylated as fibrosis severity increases [44], indicating that DNA methylation may be used as a non-invasive tool for stratifying the risk of fibrosis in NAFLD. In line with this finding, a different investigation on subjects with NAFLD revealed that DNA methylation at particular CpG dinucleotides within the human *Pparα* and *Pparγ* gene promoters can differentiate between patients with mild from those with severe fibrosis in NAFLD [45]. Later, a Turkish cohort study conducted by the same research team revealed a link between DNA methylation in the *Pparγ* promoter and fibrosis [46]. In a recent work, Hajri et al. showed that both HFD and palmitic acid alter global and *Pparγ* promoter DNA methylation, leading to significantly increased *Pparγ* expression and enhanced lipid retention in the liver, which causes NAFLD to develop [47]. Moreover, both in rat models and in NAFLD patients, it was found that *Pparγ* methylation levels significantly correlated with the severity of liver fibrosis [44,48]. It is of interest to note that *Pparγ* promoter methylation levels in plasma-free DNA were proposed as a non-invasive method to distinguish between patients with mild and severe fibrosis associated with NAFLD [48]. Another base alteration in DNA called 5-hydroxymethylcytosine (5hmC) has been found to affect DNA demethylation, which in turn affects both the activation and repression of gene transcription [49]. In this regard, an observational study by Pirola et al., suggested that the 5hmC might be involved in the pathogenesis of NAFLD by regulating liver mitochondrial biogenesis and PPARγ coactivator 1a (PGC-1α) expression [50]. 

Indirect effects of DNA methylation on *Pparγ* expression are also possible. In fact, a prior study found that, in diet-induced obese mice, methylation of hepatic interferon regulatory factor 6 (Irf6) reduces hepatic steatosis and metabolic abnormalities by transcriptionally repressing *Pparγ* [51]. It has been reported that the C-Maf inducing protein (Cmip) is associated with metabolic disorders such obesity, diabetes, and NAFLD. A further investigation demonstrated that hypomethylation of Cmip promotes its expression and facilitates the development and progression of NAFLD by activating the PPARγ-CD36 signaling pathway [52]. Furthermore, even though the findings reported here show interesting characteristics of *Pparγ* gene expression and methylation changes in relation to NAFLD, more research is required to clearly establish a causal link between the two events. Examples of DNA methylation patterns and the PPARγ pathway linked to the pathogenic feature of NAFLD are shown in Table 1.

### 2.2. Histone Modifications

An important component of the epigenetic changes that affect the transcriptional regulatory processes is the dynamic network of post-translational histone modifications. Much research has been conducted on histone methylation and acetylation as heritable epigenetic indicators for chromatin structure and function. Many enzymes control the posttranscriptional alterations of histones by interfering with particular DNA binding sites, which results in the dysregulation of specific gene expression [53]. In addition, to achieve the accurate regulation of gene expression, histone modifications frequently interact in a cooperative way with transcription factors (TFs). It has been demonstrated that an imbalance in histone modifications leads to an irregularity in transcriptional activity that is associated with the emergence of diseases such T2DM, obesity, and consequently MAFLD [54]. For instance, abnormal histone modifications have been shown to promote the development of insulin resistance and thus, NAFLD [55]. Hence, gaining a better knowledge of how cells connect histone changes to transcription factors (TFs) may open up new avenues for the identification of novel epigenetic targets and offer crucial hints for the design of functional investigations to come and prospective epigenetic treatments for NAFLD.

#### 2.2.1. Histone Methylation/Demethylation

According to a study undertaken by Kim et al., the histone H3 lysine 4 (H3K4) methyltransferase myeloid/lymphoid or mixed-lineage leukemia 4 (MLL4/KMT2D) regulates overnutrition-induced steatosis by acting as a coactivator for PPARγ2 through H3K4 methylation [56]. Further studies suggested that H3K4 and H3K9 trimethylation may contribute to hepatic steatosis and disease progression [57]. In fact, Jun et al. demonstrated that, in HFD-fed mice, aberrant histone H3K4 and H3K9 trimethylation in *Pparα* and genes involved in lipid metabolism cause hepatic steatosis [57]. Moreover, both diet-induced obese mice and NAFLD patients have considerably higher levels of the histone-lysine N-methyltransferase suppressor of variegation 3-9 homologue 2 (Suv39h2), which represses the expression of the *Sirt1* and *Pparγ* genes [58]. 

The process of histone demethylation is carried out by enzymes called histone demethylases (HDMs), which remove methyl groups from altered histones to activate or repress gene transcription. Many histone demethylases have been identified and classified into two classes: FAD-dependent amine oxidases (LSD demethylases) and Fe(II)- and α-ketoglutarate-dependent Jumonji C (JmjC) domain-containing demethylase (JMJD demethylase) [59]. PPARγ is also implicated in the regulation of adipogenic metabolism by certain demethylases. However, direct evidence that HDMs participate in the PPARγ pathway is scarce. The H3K9-specific Jumonji demethylase JMJD1A has been reported to bind to the *Pparγ* promoter, which then decrease the number of H3K9me2 marks in this region, causing modulation of hepatic stellate cells activation and liver fibrosis [60]. Inversely, increasing JMJD2B expression promoted adipogenesis and steatosis by increasing PPARγ2 expression, hepatic lipid uptake, and intracellular triglyceride accumulation [61]. Collectively, the reviewed phenotypic evidences, as summarized in t Table 2, demonstrate that histone methylation status/PPARγ axis plays important roles in the emergence of NAFLD. However, further studies are needed to comprehend the abnormalities in the histone system that may result in NAFLD through PPARγ signaling, which would greatly increase our understanding of the pathophysiology of this condition.

#### 2.2.2. Histone Acetylation/Deacetylation

The balance between acetylation and deacetylation plays a role in the regulation of gene expression. Histone acetylation is catalyzed by histone acetyltransferases (HATs), which use acetyl-CoA as a co-substrate and acetylate lysine residues on histone tails. HATs modify chromatin histones and play an important role in the epigenetic modulation of gene transcription programs. Additionally, aberrant histone modifications have been shown to contribute to the onset of IR and consequently to fatty liver disease [55]. Indeed, numerous investigations have demonstrated an association between NAFLD and changes in histone acetylation [62,63]. Moreover, studies have carefully looked into how histone (de)acetylation in *Pparγ* locus influences its expression in NAFLD. For example, a prior study revealed that histone marks on histone H3 lysine 9 acetylation are increased at *Pparγ* binding sites during adipogenesis [64]. Chromatin profiling of H3K27ac revealed that this mark is highly induced at the *Pparγ* gene locus during the course of adipogenesis and correlates with *Pparγ* gene expression [65]. It is important to keep in mind that the process of adipogenesis is accompanied by the fat synthesis, which may contribute to the occurrence and progression of NAFLD. Unfortunately, little is known about the relationship between HATs and TFs in the development of NAFLD. 

Histone deacetylases (HDACs) are known to repress gene expression by removing acetyl groups from lysine residues in the NH2 terminal tails of core histones and condensing chromatin, rendering the regions less accessible to transcription factors. A further study revealed that PPARγ deacetylation on two lysine residues (K268 and K293) induces brown remodeling of white adipose tissue and uncouples the adverse effects of TZDs from insulin sensitization [66]. Recent research has demonstrated that PPARγ deacetylation inhibits hypercholesterolemia and aging-associated atherosclerosis [67], confers the anti-atherogenic properties, and improves endothelial function in the treatment of diabetes [68]. Many aspects of mammalian development and physiology need HDAC3 [69,70]. HDAC3 genetic investigation indicates that it is a crucial regulatory component of molecular complexes that govern gene expression, which in turn affects metabolic function in the liver via numerous signaling pathways, and HDAC3 deletion in the liver affects normal metabolic homeostasis [70,71]. HDAC3 has also been demonstrated to modulate metabolism by increasing fatty acid oxidation and improving circadian histone deacetylation [72]. Interestingly, clinical studies revealed that HDAC3 expression levels in pediatric patients were correlated with overweight [73]. High levels of proinflammatory markers and insulin resistance are associated with enhanced expression of the deacetylase HDAC3 in the hepatocytes of fat-fed E3 rats that developed metabolic syndrome and in the peripheral blood mononuclear cells of T2DM patients [74]. Inhibition of HDAC3 may promote ligand-independent PPARγ activation by protein acetylation causing an increase in glucose uptake and improvement of insulin sensitivity in adipocytes [75]. 

A histone deacetylase known as Sirtuin 1 (SIRT1) has historically been associated with the control of hepatic metabolism, as well as glucose and lipid homeostasis [76]. A previous study has indicated that the deacetylating effect of SIRT1 on histone improves hepatic steatosis [77]. It was also demonstrated that better liver health is correlated with overexpression of SIRT1 in hepatocytes [78,79]. Fatty acid oxidation has been linked to SIRT1, and its deficiency negatively impacts PPARγ signaling. Interestingly, the interaction of PPARγ and SIRT1 is essential for the activation of PGC-1α. Moreover, hepatocyte-specific deletion of SIRT1 alters fatty acid metabolism and leads to hepatic steatosis and inflammation [80]. In fact, reduced levels of this protein have been observed in NAFLD patients as well as in animal models [81,82]. Parallel to this, SIRT1 suppression in the mouse liver is sufficient to cause hepatic steatosis [83], an effect that may be mediated via *Ppar-γ* and *Pparα,* the key regulators of glycolysis and lipolysis [80,84]. Adipose-specific deletion of *Sirt1* generates a hyperacetylated PPARγ state and enhanced PPARγ activity, leading to higher insulin sensitivity [84]. Collectively, these preliminary findings highlight the significance of PPARγ epigenetic regulation and histone-modifying enzymes as possible pharmaceutical targets to treat NAFLD.

### 2.3. Noncoding RNAs

ncRNAs modulate various cell biological processes in cells, including metabolism, chromatin shaping, gene transcription and translation, and posttranslational modifications. Dysregulation of these transcripts has been implicated in a variety of pathologies including NAFLD. Therefore, understanding their underlying mechanisms of action and identifying factors with which they crosstalk will make them appealing non-invasive biomarkers and therapeutic targets in fatty liver disease. As previously highlighted, peroxisome proliferator-activated receptors (PPARs) regulate lipid homeostasis and have been proposed as important regulators in the development of NAFLD and its various stages. Furthermore, their cross-regulation with ncRNAs has emerged as an additional layer of complexity in the regulatory mechanisms of several diseases, including NAFLD [85,86,87]. Thus, expanding our knowledge of the ncRNAs/PPARγ regulatory axis may help us better understand how epigenetic mechanisms contribute to the physiopathology of NAFLD and advance the process of developing potential ncRNAs/PPARγ-based therapeutics for this condition. 

#### 2.3.1. miRNAs-PPARγ Axis

miRNAs, a type of small noncoding RNAs, have been associated with the epigenetic regulation of gene expression involved in the development of steatosis and its progression to NASH, fibrosis, and HCC [88,89,90]. There is currently mounting evidence that miRNAs influence the transcription of NAFLD-related genes including those involved in PPARγ pathway [91,92]. Further research revealed that the expression of a variety of miRNAs was induced in NASH and fibrosis, and that this induction was associated with PPARγ modulation. As will become apparent in the following discussion, the mutual crosstalk between PPARγ and specific miRNAs plays substantial roles in the development of NAFLD forms. 

*miR-21:* It has been shown that miR-21 promotes hepatic lipid accumulation in part by interacting with multiple factors, including SREBP1 [93] and 3-hydroxy-3-methylglutaryl-co-enzyme A reductase [94]. Furthermore, by inhibiting the PPARα signaling pathway, miR-21 contributed to cell damage, inflammation, and fibrosis [95]. Several investigations have shown that the circulation and liver of NAFLD patients and mice models both have high amounts of miR-21 [96,97,98]. Rodrigues et al. showed that feeding miR-21 knockout animals an obeticholic acid-supplemented HFD causes a progressive decrease in steatosis, inflammation, and lipoapoptosis through PPARα upregulation and activation of the farnesoid X-activated receptor (FXR) [99]. Further research indicated that inhibition of miR-21 could alleviate steatosis by activating PPARα [95,100]. The relevance of the miR-21/PPARγ axis in NAFLD, however, remains poorly understood. According to a recent study, PPARγ regulates miR21-5p/Secreted Frizzled-related Protein 5 (SFRP5) to reduce inflammation and oxidative stress in mouse models and human tissue samples from NASH patients [101]. Mechanistically, PPARγ downregulates miR-21-5p by interacting with its promoter region, resulting in increased expression of SFRP5, an anti-inflammatory adipokine that regulates NASH progression [101]. The authors of this study suggested that the PPARγ/miR-21-5p//SFRP5 axis might be a promising target for NASH treatment. 

*miR-27:* Early studies suggested that miR-27a decreases lipid accumulation in rat HSCs and human hepatoma cells by targeting the retinoid X receptor alpha [102,103] and impairs adipocyte differentiation by targeting PPARγ [103]. A different investigation revealed that miR-27a is essential for maintaining hepatic lipid homeostasis and for the pathogenesis of NAFLD [104]. miR-27b is part of a panel of miRNAs that has been proposed for highly accurate diagnosis of NAFLD [105] and NASH in the livers of rats and zebrafish [106]. The effects of three different Western diets, a low-fat diet, a high-fat diet, and a high-fat high-fructose diet exposure, on liver PPARγ/miRNA regulation were examined, and the results revealed that a high-fat, high-fructose diet induces an intermediate stage between fatty liver and fibrosis via miR-27b-5p-induced PPARγ downregulation [107]. The miR-27b targets 3′-UTR of *Pparγ* gene [108] and contributes to the destabilization of *Pparγ* mRNA by lipopolysaccharides [109]. Disruption of endogenous miR-27b activity by a transgenic miR-27b sponge in zebrafish enhances lipid accumulation and the expression of PPARγ in the liver, leading to early onset of NAFLD and NASH [110]. Moreover, miR-27a directly targets the 3′-UTR of the *Pparγ* gene to promote proliferation of hepatocellular carcinoma cells [111,112]. Taken together, these findings support the notion that the miR-27-*Pparγ* axis may be a target for NAFLD prevention.

*miR-34:* The expression of miR-34a is altered in patients with T2DM, steatosis, NASH, and NAFLD models [113,114,115]. Several studies [116,117] found that serum levels and hepatic expression of miR-34 were higher in NAFLD/NASH patients compared to controls [116,117]. Furthermore, in patients with coronary artery disease, miR-34a expression is upregulated, which is exacerbated when the patients also have NAFLD [118]. In a mouse model, inhibiting miR-34a improved hepatic steatosis by increasing PPAR levels, which promoted lipid oxidation [119]. In an in vitro and in vivo model, the antagomir circRNA 0046366 antagonized miR-34a and restored PPAR expression, alleviating NAFLD [120]. Derdak et al. showed that inhibiting p53 transcriptional activity partially reduced steatosis, associated oxidative stress, and apoptosis in a mouse model of NAFLD by downregulating miR-34a and activating the SIRT1/PGC1α/PPARα axis [121]. Furthermore, miNA-34a and miRNA-34c promoted HSCs activation by targeting PPARγ, implying that members of the miR-34 family may be involved in liver fibrosis via PPARγ pathways [122]. 

*miR-132:* Visceral adipose tissue from obese bariatric surgery patients with biopsy-proven NASH had considerably lower levels of a panel of miRNAs expression profiles, including miR-132 [123]. However, animal models of hepatic steatosis or NASH exhibited a substantial increase in hepatic miR-132 levels and a commensurate drop in selected miR-132 targets, while miR-132 repression attenuated the steatotic phenotype [124]. miR-132 was downregulated in stellate cells from CCl4-treated animals (to induce fibrosis), which resulted in the expression of one of its targets, methyl-CpG-binding protein 2 (MeCP2). In turn, MeCP2 binds to PPARγ and enhances the development of an epigenetic repressor complex, which inhibits PPARγ transcription and, in this mouse model, causes liver fibrosis [125]. 

*miR-155:* This transcript has been found to be implicated in several inflammatory processes that control innate immunity in both alcoholic and nonalcoholic fatty liver disease [126]. Additionally, miR-155 regulates various TFs involved in lipid metabolism such as LXRα [127], PPARα and PPARγ [128], and SIRT1 [129]. The expression of miR-155 is reduced during adipogenesis in vitro, while its overexpression inhibits PPARγ and cEBPα, clearly indicating that miR-155 acts as a negative regulator of adipogenesis [130]. By suppressing PPARγ, miR-155 contributes to insulin resistance, a glucose intolerance state conferred by obese adipose tissue macrophage (ATM) exosomes [131]. Tryggestad et al. found increased expression of miR-155 by ATM in obese individuals and predicted that this would have the same effect on PPARγ and GLUT4 [132]. In a mouse model of liver fibrosis, miR-155 is induced, and PPARγ is its direct target in both naive and alcohol-treated macrophages [128], implying that PPARγ also functions as an antifibrotic gene. More interesting, several miRNAs have been identified as key regulators of hepatic steatosis. miR-30a-3p and miR-3666, for example, protect hepatocytes from steatosis by targeting PPARα and PPARγ, respectively [133,134]. Last but not least, the studies reviewed here and compiled in Table 3 affirm the relevance and complexity of the regulation of the miRNAs/PPARγ network in NAFLD emphasizing the necessity of continuing research in this promising field. 

#### 2.3.2. lncRNAs-PPARγ Axis

LncRNAs are a group of RNA molecules that have a length of more than 200 nucleotides but cannot be translated into functional proteins. These RNA transcripts have a variety of epigenetic regulatory functions in humans, including chromatin modification and remodeling, genomic imprinting, and transcriptional and translation processes [135]. lncRNAs alteration has been linked to the pathophysiology of a variety of diseases, including cancer, T2DM, CVD, and liver disease [136]. In fact, deregulation of lncRNAs has been proposed as a factor in NAFLD susceptibility [137]. There is ample evidence that lncRNAs crosstalk with nuclear receptors, including PPARs to play a pivotal role in triglyceride, cholesterol, and lipoprotein metabolism. For example, the hepatocyte-derived lncRNA (lnc-HC) regulates hepatic lipid droplets accumulation via the miR-130-3p/PPARγ pathway [138], implying that lnc-HC could be a potential therapeutic target to prevent excessive lipogenesis, lipid accumulation, and NAFLD phenotype. Through the expression of PPARγ, lncRNA AC096664.3 has a positive correlation with ATP Binding Cassette Subfamily G Member 1 expression [139]. In NAFLD animal and FFA-treated cell models, overexpression of lncRNA-H19 (H19) promotes steatosis and increases hepatic lipid accumulation and lipogenesis via the miR-130a/PPARγ axis [140]. Consistent with these results, Wang et al. revealed that the expression of H19 induces hepatic steatosis by activating the lipogenic transcription factor MLX interacting protein-like and the mammalian target of rapamycin complex 1 (mTORC1) transcriptional network in hepatocytes [141]. Another lncRNA that can affect the stage of adipogenesis, the lncRNA steroid receptor RNA activator (SRA), has also been shown to promote hepatic steatosis by repressing adipose triglyceride lipase expression [142]. SRA is known to act as a steroid receptor coactivator [143]. In adipogenesis, SRA associates with PPARγ and coactivates PPAR-dependent gene expression [144]. Aerobic exercise appears to improve lipid metabolism in obese mice via the LncSRA/p38/JNK/PPARγ signaling pathway [145]. In both humans and mice with NAFLD, the conserved Hedgehog (Hh) signaling pathway was activated [146,147]. According to one recent study, the Hh pathway plays an important role in the regulation of hepatic lipid metabolism and related diseases via direct regulation of a previously uncharacterized lncRNA termed Hedgehog signaling-induced long noncoding RNA (Hilnc) [148]. Hilnc was found to control the stability of PPARγ mRNA by directly interacting with IGF22BP2. The PPARγ signaling pathway was reduced in Hilnc knockout mice, which made them resistant to diet-induced obesity and hepatic steatosis [148]. It is interesting to note that the same research team also discovered h-Hilnc, the human homologue of Hilnc, which demonstrated a comparable role for Hilnc in lipid metabolism. These results collectively imply that understanding the epigenetic function of the lncRNA/PPARγ networks, with the most pertinent examples presented in Table 4, may improve lipid homeostasis and prevent NAFLD. Nonetheless, more research would be beneficial to pinpoint the precise role that these events played in the etiology of NAFLD.

#### 2.3.3. circRNAs-PPARγ Axis

Circular RNAs (circRNAs) are endogenous noncoding RNA molecules that have recently attracted attention due to their stable structure [149]. Recently, it was found that circRNAs sponge miRNAs by binding to miRNA response elements (MREs) [150,151]. They have been implicated in numerous important physiological and pathological processes and may serve as potential biomarkers for a variety of diseases. For instance, some circRNAs can inhibit miRNA’s function that is associated with the progression and pathophysiology of chronic liver disease [151,152,153]. Insulin resistance, which is considered to be the “first hit” in NAFLD, has been attributed to the circHIPK3 and circANKRD36-mediated sponging of miR-192-5p and miR-145, respectively [154,155]. While there is a wealth of knowledge regarding the crosstalk between PPARγ and miRNAs or lncRNAs in NAFLD, very few studies have examined the function of the circRNAs/PPARγ network. In this respect, a prior study has revealed a tight association between circRNAs with hepatic steatosis and NASH [156] and circRNA_0046367 has been found to prevent hepatic steatosis by reversing the inhibitory effect of miR-34a on PPARα by blocking miRNA/mRNA interaction with MRE [153]. Likewise, circRNA_0046366 has been shown to inhibit hepatocellular steatosis by normalizing PPARα signaling [120]. The circRNA low-density lipoprotein receptor (circLDLR) acts as a sponge for miR-667-5p to regulate SIRT1 expression in NAFLD [157]. SIRT1 is the NAD-dependent deacetylase known as a PPARγ inhibitor [158]. Together, circLDLR and SIRT1 are common targets of miR-667-5p and contribute to the development of NALFD by regulating the autophagy pathway. Recently, Lin et al. identified a novel circRNA, circRNf111 (hsa_circ_0001982, generated from exon 2 of the *RNF111* gene), whose expression is downregulated in metabolic syndrome, a risk factor for NAFLD [159]. Afterword, these authors demonstrated that circRNF111 protects against insulin resistance and fat deposition in the metabolic syndrome via controlling the miR-143-3p/IGF2R axis [159]. 

## 3. Limitations and Future Perspectives

Emerging evidence clearly indicates that alterations in PPARγ/epigenetic effectors network play a crucial role in the pathogenesis of NAFLD. These observations have sparked a renewed interest in investigating PPARγ epigenetic regulatory mechanisms as they may lead to the discovery of potential non-invasive biomarkers and therapeutic targets for NAFLD. Even though the concept is promising, there are still several limitations in the field, and these will be pointed out. (i) While the studies discussed above provided evidence that epigenetic factors contribute to the dysregulation of PPARγ in NAFLD, the impact of the genetic determinants of this TF was not considered in this work. This is challenging because variants in the *Pparγ* gene have been linked to metabolic disorders such as atherosclerosis, diabetes, obesity, and NAFLD. In fact, a missense Pro12Ala substitution in the *Pparγ2* gene (rs1801282) has been investigated in relation to NAFLD risk in several ethnic groups, and those who carry the *Pparγ2* Ala allele polymorphism showed a protective effect against NAFLD [160,161]. Additionally, the SNP rs3856806 (also referred to as C161T or C1431T) in the *Pparγ* gene increases NAFLD susceptibility through the adiponectin pathway [162,163]. Therefore, the fundamental question that remains unanswered is to what extent these genetic variations drive the potential interactions between PPARγ and epigenetic regulators. (ii) Another significant limitation in developing therapeutics for NAFLD/NASH is a lack of suitable and validated preclinical models that mimic the pathophysiology of human disease [164,165]. Despite the fact that molecular epigenetic modifications such as DNA methylation are apparent across species, there are no relevant preclinical models to investigate the role of epigenetic mechanisms in NAFLD. This drawback emphasizes the urgent need to develop newer and more pertinent epigenetic models that offer insightful information about the pathophysiology of NAFLD and promote their successful implementation in real-world human therapeutic settings. In this respect, epigenome editing is gaining ground as a promising strategy to reverse aberrant epigenetic drivers of disease. In fact, there have been recent attempts to enable gene expression reprogramming by targeting epigenetic editing of locus-specific sites using DNA technology to generate these models. For example, to attenuate fibrosis in a mouse model, Xu et al. successfully demonstrated targeted demethylation of the *Rasal1* and *Klotho* genes by in vivo lentiviral delivery [166]. Similar to this, Horii et al. generated a mouse model for epigenetic disorders through targeted epigenome demethylation [167]. Consequently, using epigenome editing technologies in a preclinical model could lead to a better knowledge of how epigenetic expression is controlled and the development of new therapeutic tools. (iii) The tight bidirectional communication between adipose tissue and liver is known to involve multiple factors including lipids, adipokines, and secreted molecules that can affect the expression key TFs such as SREBP1c and PPARγ, thus leading to enhanced pathological process of NAFLD. In addition, inflammatory signals and immune mediators can interact with chromatin to influence changes in the epigenetic landscape. However, it is unclear how adipose tissue/liver axis dysfunction affects PPARγ/epigenetic mechanisms via immune and inflammatory factors. (iv) There is an ambiguity as to whether PPARγ is in favor or against the development of NAFLD. As was previously mentioned, PPARγ is abundantly expressed in adipose tissue, where it is crucial for the control of adipogenesis, fat storage, and glucose metabolism. Several clinical trials have examined the ability of TZDs to lower NAFLD/NASH by targeting PPARγ [168]. This decrease could be attributed to PPARγ activation in adipose tissue, which promotes adipogenesis while inhibiting lipolysis, lowering the amount of fatty acids entering the liver [169]. In fact, adipogenesis is known to be a physiological process that improves tissue’s ability to safely store lipids and avoid lipotoxicity in peripheral organs such as the liver. PPARγ expression is low in healthy liver, but once activated, it can provide several benefits such as protection from oxidation, inflammation, fibrosis, fatty liver [170], and insulin sensitizing activity [27]. Paradoxically, there is a strong correlation between the onset of NAFLD and hepatocyte-specific PPARγ expression. Furthermore, hepatic PPARγ expression is strongly induced in NAFLD patients and experimental models [28,171]. Therefore, this ambiguity must be resolved. Particular focus should be placed on the potential crosstalk between PPARγ pathways and epigenetic factors and how this might be fine-tuned in different tissues and contexts. (v) Another important pathway involved in the pathogenesis of NAFLD is the tripartite interaction of adipose tissue, gut, and liver [172]. It is unclear what role PPARγ and epigenetic mechanisms might play in regulating such an organ-organ connection. In the long run, exploring the gut-liver-adipose tissue axis, which integrates epigenetic and TFs mechanisms, may therefore lead to better understanding of the pathogenesis of liver disease as well as new treatment options.

## 4. Conclusions

**Figure 2:**
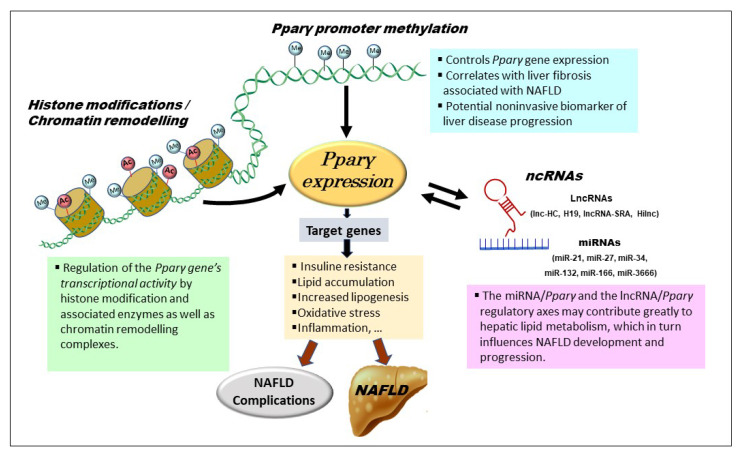
Epigenetic regulatory mechanisms of PPAR*γ* in NAFLD. *Pparγ* promoter methylation changes alter PPARγ expression and, as a result, contribute to NAFLD development. Histone modification in the vicinity of *Pparγ* promoter can regulate PPARγ expression and lipogenesis. The expression of PPARγ and its target genes is regulated by chromatin remodeling at the *Pparγ* locus. Many ncRNAs, primarily miRNAs and lncRNAs, can influence PPARγ expression via crosstalk regulation. Epigenetic modifiers are themselves subject to regulation by environmental stimuli and genetic factors. Ac—acetylation; Me—methylation; ncRNAs—noncoding RNAs.

The findings from numerous human and animal studies reviewed above and recapitulated in Figure 2 clearly support the notion that dysregulation in the crosstalk between PPARγ singling and epigenetic effectors plays a significant role in the pathophysiology of NAFLD and its progression stages. Because NAFLD is likely to affect more than one organ, this may be a compelling rationale to investigate the mechanisms governing the functional association and interplay of PPAR and epigenetic regulators, not only in the liver but also in other organs and systems implicated in NAFLD. It is hoped that this knowledge will shed more light on the pathogenesis of the condition and may lead to the identification of novel epigenetic targets and signaling pathways that may hold crucial information for the development of functional studies in the future and potential epigenetic treatments for NAFLD.

## Figures and Tables

**Table 1:** Epigenetic regulation of PPARγ by DNA methylation in NAFLD.

Epigenetic Change	Biological Effect	Reference
*Pparα/Pparγ/* methylation	DNA methylation of *Pparα and Pparγ* can distinguish between mild and severe NAFLD-associated fibrosis	[45]
*Pparγ* promoter methylation	HFD and palmitic acid alter global and *Pparγ* promoter DNA methylation, resulting in *Pparγ* expression and enhanced lipid retention in the liver, which leads to the development of NAFLD	[47]
*Pparγ* promoter methylation	*Pparγ* promoter hypermethylation levels in plasma-free DNA could be used as a non-invasive method to differentiate between NAFLD patients with mild and severe fibrosis	[48]
*Pparγ* promoter methylation	Methylation levels of *Pparγ* correlate with liver fibrosis in rat model as well as in NAFLD patients	[44,48]

Abbreviations: HFD—high-fat diet; miRNAs—microRNAs; NAFLD—nonalcoholic fatty liver disease; NASH—nonalcoholic steatohepatitis; PPARγ—peroxisome proliferator-activated receptor gamma.

**Table 2:** Epigenetic regulation of PPARγ through histone methylation mechanisms.

Epigenetic Effector	Biological Effect	Reference
MLL4	Murine steatosis caused by excessive feeding is regulated by the histone H3 lysine 4 methyltransferase MLL4/KMT2D via PPARγ2	[56]
Suv39h2	SUV39H2 expression in hepatocytes, mice, and human livers is induced by pro-NASH stimuli, and thus contributes to NASH pathogenesis by suppressing *Pparγ* and *Sirt1* expression.	[58]
JMJD1A	JMJD1A promotes PPARγ expression by regulating the demethylation of *Pparγ* gene and thus inhibit HSCs activation and fibrosis	[60]
JMJD2B	JMJD2B promotes the development of hepatic steatosis by upregulating PPARγ2 and steatosis target genes.	[61]

Abbreviations: JMJD2B—JumonjiC (JmjC) domain containing histone lysine demethylase; HSCs—hepatic stellate cells; MLL4—histone H3-lysine 4 (H3K4)-methyltransferase; NAFLD—nonalcoholic fatty liver disease; NASH—nonalcoholic steatohepatitis; PPARγ—peroxisome proliferator-activated receptor gamma; Suv39h2—histone H3K9 methyltransferase suppressor of variegation 39 homolog 2.

**Table 3:** Relevant miRNAs shown to be associated with PPARγ in NAFLD and its complications.

miRNA	Target or Pathway	Pathophysiological Processes	References
miR-21	PPARγ/SFR5	PPARγ prevents inflammation and oxidative stress in mouse models and human tissue samples from NASH patients by modulating the miR21-5p/SFRP5 pathway	[101]
miR-27	PPARγ	Disrupting endogenous miR-27b activity in Zebrafish causes lipid accumulation and increased PPARγ expression in the liver, resulting in the early onset of NAFLD and NASH	[110]
miR-34	PPARγ	By targeting PPARγ, miR-34a/c activation may be associated with liver fibrosis	[122]
miR-132	PPARγ/MeCP/EZH2	miR132/MeCP2/EZH2 axis is involved in the regulation of liver fibrosis	[125]
miR-155	PPARγ	miR-155 contained in obese ATM-exosomes, contributes to insulin resistance and glucose intolerance via a mechanism that is most likely related to direct suppression of its target gene P*parγ*	[131]
miR-3666	PPARγ	miR-3666 inhibits the development of hepatic steatosis by negatively regulating PPARγ	[134]

Abbreviations: ATM-Exos—adipose tissue macrophages containing exosomes; MeCP2—methyl-CpG binding protein 2; miRNAs—microRNAs; NAFLD—nonalcoholic fatty liver disease; NASH—nonalcoholic steatohepatitis; PPARγ—peroxisome proliferator-activated receptor gamma; SFRP5—Secreted Frizzled Related Protein 5.

**Table 4:** Relevant lncRNAs shown to regulate PPARγ in NAFLD and associated complications.

LncRNA	Targeted or Pathway	Pathophysiological Processes	References
Lnc-HC	miR-130-3p/PPARγ	LncRNA regulates hepatic lipid droplets accumulation via miR-130-3p/PPARγ pathway, which may help to prevent NAFLD	[138]
H19	miR-130a/PPARγ	H19 promotes hepatic lipogenesis and the progression of NAFLD via miR-130a/PPARγ axis	[140]
LncRNA-SRA	PPARγ	LncRNA-SRA promotes hepatic steatosis by repressing the expression of adipose triglyceride lipase	[141]
Hilnc	IGF22BP2/PPARγ	The loss of Hilnc prevents diet-induced hepatic steatosis by inhibiting PPARγ	[148]

Abbreviations: IGF2BP2—Insulin-like growth factor 2 mRNA-binding protein 2; lncRNAs—long noncoding RNAs; NAFLD—nonalcoholic fatty liver disease; NASH—nonalcoholic steatohepatitis; PPARγ—peroxisome proliferator-activated receptor gamma; SRA—steroid receptor RNA activator.

## Data Availability

Not applicable.
